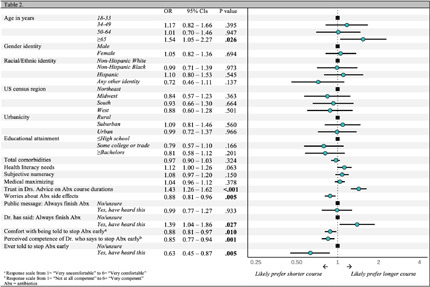# US Adults’ Perspectives on Antibiotic Durations and Adherence to Therapy for Bacterial Respiratory Infections

**DOI:** 10.1017/ash.2025.217

**Published:** 2025-09-24

**Authors:** Alistair Thorpe, Rachael A. Lee, Julia E. Szymczak, Madeline Farrell, Angela Fagerlin, Valerie M. Vaughn

**Affiliations:** 1 University of Utah School of Medicine; 2 University of Alabama at Birmingham; 3 N/A; 4 Salt Lake City VA Informatics Decision-Enhancement and Analytic Sciences (IDEAS) Center for Innovation

## Abstract

**Background:** Calls within the clinical community for revising guidance on the appropriate durations of antibiotic therapy (i.e., shorter is better) and adherence (i.e., no longer advising to always finish a course), reflect important gains in evidence-based prescribing. However, changing medical guidance can have negative public effects (e.g., frustration, distrust, and disengagement) when not communicated in ways that resonate with patients. To inform efforts to effectively communicate evolving evidence on appropriate antibiotic use, we examined US adults’ perceptions and preferences regarding antibiotic durations and adherence. **Methods:** From March to April 2024, we invited US adults, aged ≥18 years, to an online survey about antibiotics. Question topics included durations of antibiotic therapy, adherence to a prescribed course of antibiotics, and demographic characteristics. **Results:** Table 1 shows the characteristics of the 1,476 respondents [completion=89%]. Most respondents reported they preferred to take a longer course of antibiotics (≥7 days) than a shorter one (3-5 days) for a bacterial respiratory infection (60.4% vs. 39.5%) and rated longer courses as both safer and more effective (Table 2). In open-text questions, respondents who preferred shorter courses described a general aversion to medication and concerns about side effects and resistance, whereas those who preferred longer courses saw them as familiar and a ‘better safe than sorry’ approach, associating longer durations with greater efficacy. In addition, 88.4% of respondents agreed that ‘it is important to always finish a prescribed course of antibiotics, even if you start to feel better’ and had either been told this by a medical professional (76.3%) or seen this guidance in a public health message (61.2%). Conversely, only 17.5% said they had ever been told they could stop taking antibiotics early. Preference for longer antibiotic courses was associated with older age, trusting their doctor’s advice about antibiotic therapy durations, having been told by their doctor to ‘always finish a course of antibiotics’, less worry about antibiotic side effects, discomfort about potentially being asked by a clinician to stop taking antibiotics when they start to feel better, and perceiving the clinician suggesting that as less competent. **Conclusions:** Many US adults prefer longer durations of antibiotic therapy for respiratory infections than are likely necessary. Almost all survey respondents believed it important to always finish a course and many were uncomfortable with advice to the contrary. These findings highlight the need for evidence-based communication strategies for aligning US adults’ antibiotic duration and adherence preferences with current guidance.

**Figure:**
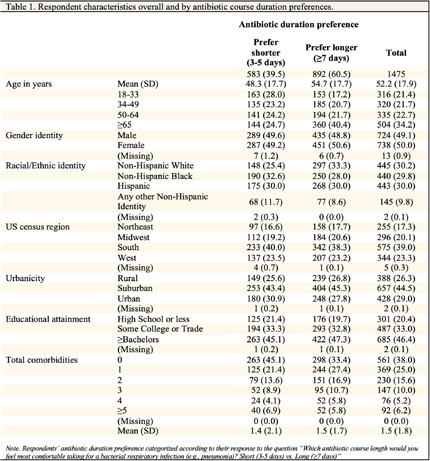

**Figure:**